# Crosstalk between chromatin structure, cohesin activity and transcription

**DOI:** 10.1186/s13072-019-0293-6

**Published:** 2019-07-22

**Authors:** Douglas Maya-Miles, Eloísa Andújar, Mónica Pérez-Alegre, Marina Murillo-Pineda, Marta Barrientos-Moreno, María J. Cabello-Lobato, Elena Gómez-Marín, Macarena Morillo-Huesca, Félix Prado

**Affiliations:** 1 Department of Genome Biology, Andalusian Molecular Biology and Regenerative Medicine (CABIMER), CSIC-University of Seville-University Pablo de Olavide, Seville, Spain; 2 Genomic Unit, Andalusian Molecular Biology and Regenerative Medicine Center (CABIMER), CSIC-University of Seville-University Pablo de Olavide, Seville, Spain; 3 Present Address: Department of Biochemistry, University of Oxford, Oxford, UK; 4 Present Address: Division of Cancer Sciences, Manchester Cancer Research Center, University of Manchester, Manchester, UK

**Keywords:** Chromatin, Cohesin, Transcription, Scc1

## Abstract

**Background:**

A complex interplay between chromatin and topological machineries is critical for genome architecture and function. However, little is known about these reciprocal interactions, even for cohesin, despite its multiple roles in DNA metabolism.

**Results:**

We have used genome-wide analyses to address how cohesins and chromatin structure impact each other in yeast. Cohesin inactivation in *scc1*-*73* mutants during the S and G2 phases causes specific changes in chromatin structure that preferentially take place at promoters; these changes include a significant increase in the occupancy of the − 1 and + 1 nucleosomes. In addition, cohesins play a major role in transcription regulation that is associated with specific promoter chromatin architecture. In *scc1*-*73* cells, downregulated genes are enriched in promoters with short or no nucleosome-free region (NFR) and a fragile “nucleosome − 1/RSC complex” particle. These results, together with a preferential increase in the occupancy of nucleosome − 1 of these genes, suggest that cohesins promote transcription activation by helping RSC to form the NFR. In sharp contrast, the *scc1*-*73* upregulated genes are enriched in promoters with an “open” chromatin structure and are mostly at cohesin-enriched regions, suggesting that a local accumulation of cohesins might help to inhibit transcription. On the other hand, a dramatic loss of chromatin integrity by histone depletion during DNA replication has a moderate effect on the accumulation and distribution of cohesin peaks along the genome.

**Conclusions:**

Our analyses of the interplay between chromatin integrity and cohesin activity suggest that cohesins play a major role in transcription regulation, which is associated with specific chromatin architecture and cohesin-mediated nucleosome alterations of the regulated promoters. In contrast, chromatin integrity plays only a minor role in the binding and distribution of cohesins.

**Electronic supplementary material:**

The online version of this article (10.1186/s13072-019-0293-6) contains supplementary material, which is available to authorized users.

## Background

The first level of structural organization of chromosomes is the chromatin fiber, in which DNA is assembled into regularly spaced nucleosomes through the coordinated activity of histone chaperones, chromatin assembly factors and nucleosome modifiers and remodellers. Assembly of replicated DNA is coupled to the replication fork and occurs by the deposition of both newly synthesized and parental histones, which are distributed randomly between the sister chromatids [1]. In addition, replication-independent mechanisms of nucleosome assembly reset the chromatin changes induced by processes like transcription or DNA repair during the cell cycle [2, 3].

Chromatin fiber permits several levels of compaction of DNA into the nucleus, depending on the nuclear functional state or the cell cycle phase. Although the exact molecular structure behind compaction is still unclear, current models suggest that the packaging of chromatin requires dynamic and regulated interactions between irregularly folded fibers of nucleosomes, leading to a meshwork of intramolecular contacts [4]. The compaction, dynamics and regulation of this network rely mostly on the coordinated activities of topological machineries: cohesins, condensins and topoisomerases [5]. Whereas topoisomerases change the DNA supercoiling by cutting DNA molecules and re-sealing them in a new topological state [6], cohesins and condensins are ring-shaped molecules able to hold together distant chromatin fragments, thus compacting chromosomes [7].

In budding yeast, cohesins are loaded at chromatin by the cohesin loader complex Scc2/Scc4 during G1 and early S phase in a non-stable conformation that promotes topological and non-topological interactions (depending on whether or not cohesin entraps chromosomal DNA inside its ring) through dynamic turnover [8–10]. After loading, cohesins are moved away by the transcriptional machinery and accumulate preferentially at intergenic regions (IGRs) [11–14]. A fraction of cohesins becomes cohesive during S phase by topologically entrapping the two sister chromatids [15–18] and remains stably bound until its degradation in mitosis [19] (reviewed in [20, 21]). Although essential, sister chromatid cohesion is not the only function of cohesins, and multiple roles in DNA compaction, transcription regulation, DNA repair and DNA replication have been revealed in the past few years [22–25]. Remarkably, Scc2/Scc4 has been reported to have a role in RSC-mediated chromatin remodeling and transcription [26]. Specifically, the chromatin remodeling complex RSC recruits Scc2/Scc4 to specific promoters, where the cohesin loader helps to maintain the nucleosome-free region (NFR) for transcription activation [26]. RSC also interacts with and is required for cohesin loading [27] through a mechanism that involves direct interactions between RSC and both cohesin and Scc2/Scc4 [28]. However, cohesins and cohesin loaders accumulate at non-overlapping peaks along the genome after their loading [12]. Therefore, it is unclear whether or not cohesins take part in chromatin remodeling and/or transcription in yeast.

Nucleosomes seem to have an inhibitory effect on cohesin binding. The Snf2-related complexes RSC and Irc5 in yeast, and SNF2h in humans are required for cohesin binding to chromosomes [27, 29, 30]. Importantly, although the chromatin remodeling activity of RSC is not necessary for cohesin loader recruitment, an ATPase dead RSC complex is defective for cohesin loading; accordingly, nucleosomes interfere with cohesin loading in vitro [28]. The distribution of cohesins is also associated with NFRs [31], although this preference might be an indirect consequence of their accumulation at IGRs [11, 13, 32], which contain NFRs [33, 34].

To address the connection between chromatin structure and cohesin function, we have now studied whether disrupting one affects the other. For this, we allow cells to progress from G1 to mitosis under conditions of histone depletion and/or lack of cohesin activity. Defective chromatin positioning by histone depletion had little or only region-specific effects on the accumulation and distribution of the major cohesin peaks. In contrast, the lack of cohesin activity affected the primary structure of chromatin at specific genomic regions, preferentially promoters. Critically, genome-wide analyses revealed a major role for cohesins in transcription regulation that is associated with the promoter chromatin architecture and location of the regulated genes.

## Results

### Cohesins contribute to structuring chromatin

To analyze what impact, if any, cohesins have on chromatin structure, we performed high-throughput sequencing of MNaseI-digested chromatin (MNase-seq) followed by dynamic analysis of nucleosome position and occupancy by sequencing (DANPOS) [35]. This approach allows nucleosomes to be mapped along the whole genome and categorizes the altered ones as changed in occupancy (measure of nucleosome density), position shift and fuzziness (degree of nucleosome deviation from its preferred position in a population). The experiment was performed with cells that express a thermosensitive allele of *SCC1* (*scc1*-*73*). The α-kleisin subunit Scc1 forms the tripartite ring-like structure with the SMC (structural maintenance of chromosomes) subunits Smc1 and Smc3 in the cohesin complex. The *scc1*-*73* allele encodes a mutant protein (S525 N) that loses its ability to interact with Smc1/Smc3 at 37 °C and thereby causes cohesin inactivation [8, 36].

**Fig. 1:**
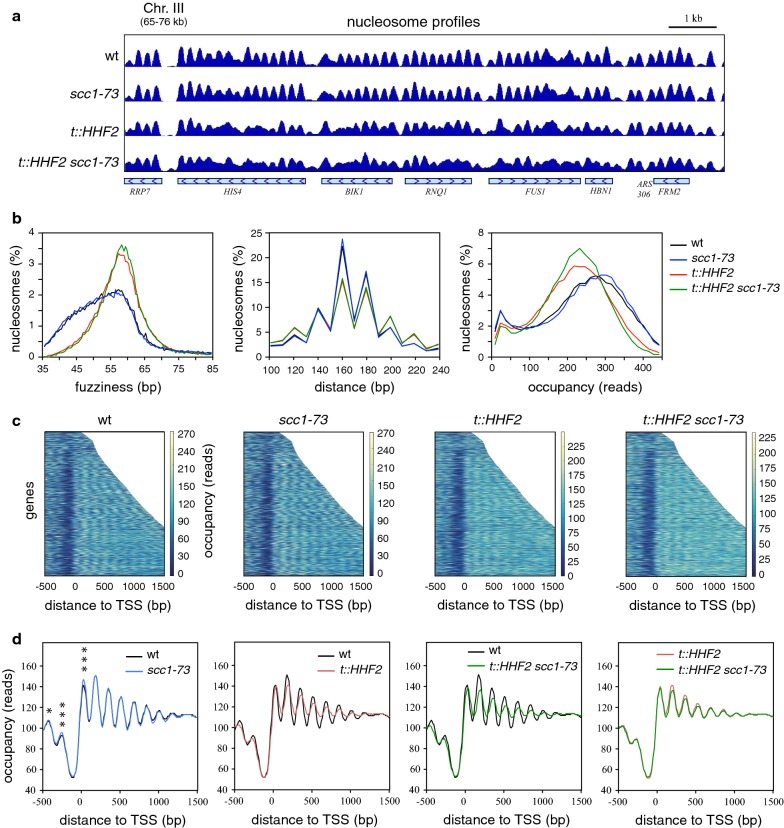
Genome-wide nucleosome profiles in cohesin and histone deposition mutants. **a** Representative nucleosome profile by MNase-seq of wild type, *scc1*-*73*, *t::HHF2* and *t::HHF2 scc1*-*73* cells synchronized in G1 and released until metaphase in nocodazole-containing (15 μg/ml) medium under conditions of restrictive temperature (37 °C) and histone depletion. **b** Histone depletion affects nucleosome fuzziness, distance and occupancy. Distribution of nucleosome fuzziness scores, neighboring distances and occupancy periodicities were determined by DANPOS analyses. **c** and **d** Heat map (**c**) and occupancy profile (**d**) of nucleosomes for all yeast genes aligned relative to the transcription start site (TSS). The occupancy of nucleosomes − 1 and + 1 was specifically increased in *scc1*-*73* cells. Statistically significant differences in occupancy at the peak of nucleosomes − 2 to + 5 between the wild type and the *scc1*-*73* mutant are shown (paired two-tailed Student’s *t* test; *, *p* < 0.05; ***, *p* < 0.001)

Cells were grown and synchronized in G1 at 26 °C, released at 37 °C and arrested in metaphase with nocodazole. This strategy ensures analysis of cells that have completed replication without cohesin activity and that have accumulated at the same cell cycle stage. The global profile of nucleosomes was similar in both *scc1*-*73* and wild-type cells (Fig. 1a). Accordingly, the lack of cohesin activity did not affect the distribution of nucleosome fuzziness scores, neighboring distances or occupancy periodicities (Fig. 1b). However, some changes were observed when nucleosome occupancy was aligned for all genes relative to the transcription start site (TSS). Thus, although the pattern of nucleosome positioning at both sides of the promoter-associated NFR was apparently unaffected (Fig. 1c), the lack of cohesin activity caused a significant increase in the occupancy of nucleosomes − 1 and + 1, but not in the occupancy of the nucleosomes in the gene body (+ 2 to + 5) (Fig. 1d, asterisks). In addition, DANPOS analysis revealed an elevated number of altered nucleosomes in *scc1*-*73* cells as compared to wild-type cells (~ 1.1% of total nucleosomes; Table 1). Most alterations affected the nucleosome occupancy, with a similar number of nucleosomes displaying either higher or lower occupancy than in wild-type cells. Further analysis of the altered nucleosome distribution in the genome showed that they were enriched in IGRs, tRNA genes and telomeres; averagely distributed in the pericentric chromatin and rDNA; and had a less than average distribution in the ORFs (Additional file 1: Table S1 and Additional file 2: Table S2). We conclude that cohesins contribute to establish the primary structure of chromatin at specific DNA regions, preferentially promoters.

**Table 1:** Number of altered nucleosomes (in position, fuzziness and/or occupancy) upon histone depletion (in *t::HHF2* cells) or cohesin inactivation (in *scc1*-*73* cells) as compared to the profile of nucleosomes in wild-type cells

	*t::HHF2*	*scc1*-*73*	*t::HHF2 scc1*-*73*
Altered nucleosomes	4431*	768	6151*
Position shift	319	35	389
Fuzziness change	1516	77	2093
Occupancy change	3989	703	5618
Occupancy increase	1304	375	2080
Occupancy decrease	2685	328	3538

* The percentage of altered nucleosomes between *t::HHF2* cells (6.68%) and *scc1*-*73 t::HHF2* cells (9.28%) was statistically different according to a two-tailed Chi-square test (*p *< 0.001)

### Lack of cohesin activity has a major effect in transcription that is linked to specific chromatin structure at promoters

**Fig. 2:**
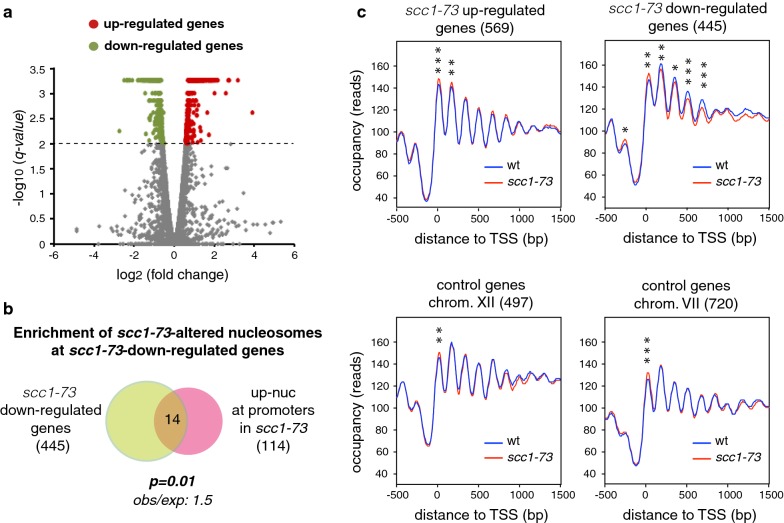
Transcription downregulation in *scc1*-*73* cells is associated with chromatin changes. **a** Volcano plot of altered genes in *scc1*-*73* cells relative to wild-type cells (1014 out of 6692 genes; ~ 15%). Changes in gene expression (log_2_) are plotted against *q*-values (–log_10_). Genes with a *q*-value < 0.01 are shown in green for downregulated (445) and in red for upregulated (569). **b** Overlap between downregulated genes, and genes with promoters that contain nucleosomes with higher occupancy (up-nucleosomes), in *scc1*-*73* cells as compared to wild-type cells. The number of genes in each case is indicated in parentheses. The probability of producing the given overlap based on random distributions was generated using a hypergeometric test. The rate between the observed and expected frequencies of common genes is also shown. **c** Nucleosome occupancy profiles of the *scc1*-*73* up- and downregulated genes, aligned relative to their TSS. Non-affected genes from chromosomes VII and XII were used as control groups. The number of genes of each group is indicated in parentheses. Statistically significant differences in occupancy at the peak of nucleosomes − 2 to + 5 between the wild-type cells and the *scc1*-*73* mutants are shown (paired two-tailed Student’s *t* test, whereby one, two and three asterisks indicate *p* values of < 0.05, < 0.01 and < 0.001, respectively)

As the effect of *scc1*-*73* was more prominent on the chromatin of IGRs and the occupancy of nucleosomes − 1 and + 1, we asked if those chromatin changes are associated with alterations in the pattern of transcription regulation. We synchronized wild-type and *scc1*-*73* cells in G1 and then released them into fresh medium until metaphase under restrictive conditions, to compare their genome-wide transcription profiles by RNA-seq under the same conditions that were used to study their chromatin structure. The absence of cohesin activity from G1 to metaphase in the *scc1*-*73* mutant yielded an elevated number of misregulated genes, with 445 downregulated genes and 569 upregulated genes (*q value* < 0.01; 1.41-fold cutoff relative to wild type); this represents ~ 15% of all yeast genes. (Noncoding RNAs were not affected.) Of these, 255 genes (~ 4%; 91 downregulated and 164 upregulated) showed a > 2-fold change (Fig. 2a and Additional file 3: Table S3). These genes showed a significant overrepresentation of gene ontology biological process terms related to RNA and amino acids metabolism and stress responses (Additional file 3: Table S3).

Next, we asked whether gene regulation by cohesins is associated with chromatin alterations. Misregulated genes upon cohesin inactivation in the *scc1*-*73* mutant were not associated with DANPOS-defined altered nucleosomes, indicating that these alterations were not caused by defective transcription. The only exception was a slight enrichment in nucleosomes with higher occupancy at the promoters of the downregulated genes (Fig. 2b). In line with this, the increase in the occupancy of nucleosome − 1 remained significant in the *scc1*-*73* downregulated (but not upregulated) genes, even though it was lost in control groups (chromosomes VII and XII) with a similar number of genes (Fig. 2c). (Note that the number of genes analyzed in Fig. 1d was ~ 10 times higher than that in Fig. 2c.) The downregulated genes in *scc1*-*73* cells were also characterized by significant changes at the nucleosomes downstream of the promoter, which might result from defective transcription. In contrast to these changes, the increase in the occupancy of nucleosome + 1 was also detected in control groups of transcriptionally unaffected genes (Fig. 2c, chromosomes VII and XII), indicating that it was transcription independent.

We next addressed whether the effects that a lack of cohesin activity had on transcription were associated with the promoter chromatin architecture. In yeast, gene promoters can be grouped according to their chromatin structure. One of these classifications distinguishes genes with occupied-proximal nucleosome (OPN) and depleted-proximal nucleosome (DPN) promoters, depending on the absence or presence of an NFR just upstream of nucleosome + 1 [37]. A similar analysis defines a higher number of genes as “open” or “closed” depending on the presence or absence of a clear NFR at the promoter [38]. Notably, *scc1*-*73* downregulated genes were highly enriched in OPN and “closed” genes (Table 2).

**Table 2:** Down- and upregulated genes in *scc1*-*73* cells are associated with a specific chromatin structure at promoters and cohesin peaks, respectively

	OPN promoter (544)	DPN promoter (494)	Closed promoter (1646)	Open promoter (3586)	FN promoter (1953)	SN promoter (3066)	RSC-binding promoter (239)	Scc2- binding promoter (199)	TATA-containing (1090)	SAGA-dominated (577)	TFIID-dominated (5130)	Mediator-dominated (315)*	NuA4- dominated (500)*	SWR1- dominated (462)	Scc1-binding promoter (2417)
*scc1*-*73* Up-reg. (569)															
Overlap.	57	51	149	373	222	300	15	16	85	50	467	29	92	41	298
Obs/exp (*p* val)	1.2 (0.015)	1.2 (0.021)	1.06 (0.026)	**1.22** **(1.3e−10)**	**1.34** **(3.5e−8)**	**1.15** **(9e−5)**	0.74 (0.045)	0.95 (0.1)	0.92 (0.03)	1.02 (0.06)	**1.07** **(2.1e−4)**	1.08 (0.07)	**2.25** **(1e−14)**	1.04 (0.06)	**1.45** **(4.9e−17)**
*scc1*-*73* Down-reg. (445)															
Overlap.	93	27	153	246	212	202	36	15	166	103	307	53	25	48	136
Obs/exp (*p* val)	**2.56** **(8.8e−19)**	0.83 (0.017)	**1.4** **(4.3e−7)**	1 (0.03)	**1.63** **(7.7e−18)**	0.99 (0.039)	**2.26** **(1.5e−6)**	1.13 (0.095)	**2.29** **(4.2e−29)**	**2.68** **(2e−22)**	***0.9*** **(2.7e−5)**	**2.53** **(9.8e−11)**	0.78 (0.02)	**1.56** **(5e−4)**	0.85 (1.6e**−**3)

The number of genes in each case is indicated in parentheses. The probability of producing the given overlapping if their distributions were random was generated using a hypergeometric test. The rate between the observed and expected frequencies of common genes is also shown. Significantly higher (underlined) and lower (italics) differences (*p* value < 0.001) are highlighted in bold.

*Only downregulated genes are analyzed. See Additional file 4: Table S4 for list of genes and references

More recent and exhaustive studies involving deep sequencing of MNaseI-treated DNA at different degrees of digestion uncovered two groups of promoters, defined according to the stability of nucleosome − 1: (*i*) promoters with a nuclease-resistant nucleosome (stable nucleosome, SN), which have a constitutive NFR of less than 150 bp, and (*ii*) promoters with a nuclease-sensitive nucleosome (fragile nucleosome, FN), which is removed through the action of transcription factors; these factors, together with the chromatin remodeling complex RSC, lead to an NFR of more than 150 bp upon transcription activation [39, 40]. Notably, *scc1*-*73* downregulated genes are highly enriched in FN promoters (Table 2) and accordingly in promoters with RSC/nucleosome complexes [41] (Table 2). Finally, we asked if *scc1*-*73* downregulated genes were preferentially associated with Scc2/Scc4-regulated genes, as RSC-mediated chromatin remodeling and transcription activation requires Scc2/Scc4 promoter binding [26]. We did not find a preferential association between Scc2-binding genes and *scc1*-*73*-misregulated genes (Table 2). Therefore, transcription activation by cohesins seems to be linked to specific chromatin structure at promoters. These results, together with a preferential increase in the occupancy of nucleosome − 1 of *scc1*-*73*-downregulated genes (Fig. 2c), suggest that cohesins promote transcription activation by helping RSC to form the NFR.

Approximately 20% of yeast genes contain a TATA box [42]. This element is enriched in the OPN genes and underrepresented in the DPN genes [37], and accordingly, the TATA box was highly enriched among the downregulated genes in *scc1*-*73* cells (Table 2). Early studies analyzing steady-state RNA levels showed that TATA-containing genes preferentially use the SAGA complex rather than the general transcription factor TFIID [42]. Similar studies have revealed a predominant role for TFIID (~ 90% of genes) and a more restricted role for SAGA (~ 10% of genes) [43]. More recent analyses have also shown that the Mediator complex is particularly important for the expression of TATA-containing, SAGA-dominated genes, whereas the histone acetyltransferase complex NuA4 is preferentially associated with TFIID [44, 45]. As expected from these associations, the set of downregulated genes in *scc1*-*73* cells were dominated by the Mediator and SAGA and not by NuA4 and TFIID; in addition, they were enriched in SWR1-dominated genes [46] (Table 2). In marked contrast with *scc1*-73-downregulated genes, *scc1*-73-upregulated genes were enriched in genes with “open” promoters and dominated by TFIID and NuA4 (Table 2). Even though these associations may reflect different mechanisms of gene regulation, they connect the role of cohesin in gene expression with specific configurations of transcriptional regulators at promoters.

### The lack of cohesin activity and histone depletion leads to similar occupancy changes in a subset of nucleosomes

Our group has previously reported that the increased accessibility to MNaseI of the centromeric chromatin at *CEN3* that can be observed in histone-depleted cells is partially suppressed by cohesin inactivation, suggesting that cohesins could contribute to the loss of chromatin integrity associated with defective histone deposition [47]. To further understand the function of cohesins in chromatin organization, we analyzed the genome-wide effects of the *scc1*-*73* allele in cells that express histone H4 from the doxycycline (dox)-regulatable *tet* promoter (*t::HHF2*) [48]. For this, *t::HHF2* and *t::HHF2 scc1*-*73* cells were released together with *scc1*-*73* and wild-type cells from G1 under conditions of histone depletion and restrictive temperature (Fig. 1). Cells depleted of histone H4 during S phase displayed a severely altered nucleosome profile in metaphase (Fig. 1a), which was especially enriched in nucleosomes with reduced occupancy (Table 1). Histone depletion caused a global increase in fuzziness and a wider distribution of both nucleosome occupancy and distance between adjacent nucleosomes (Fig. 1b). The overall loss of nucleosome positioning became particularly evident when nucleosome occupancy was aligned for all genes relative to the TSS, where the precise pattern of nucleosome positioning at both sides of the promoter-associated NFR was lost following histone depletion (Figs. 1c, d). These results are in accordance with a previous study showing that defective histone supply after H3 depletion strongly affects chromatin integrity genome-wide [49].

The double-mutant *t::HHF2 scc1*-*73* displayed a large number of chromatin perturbations (Fig. 1a, d). This loss of chromatin integrity at *t::HHF2* cells was confirmed at three different regions by indirect end labeling of MNaseI-treated cells (Additional file 8: Fig. S1). Importantly, the lack of Scc1 activity increased the number of genome-wide altered nucleosomes in histone-depleted cells (Table 1; compare *t::HHF2 scc1*-*73* with *t::HHF2*), consistent with an additive effect of the lack of histones and cohesin activity. Thus, the partial suppression of nucleosome alterations at *CEN3* described previously was specific for that locus [47]. We could not check these changes in our MNase-seq experiments because the centromeric nucleosomes were not detected (as previously reported in similar studies [49, 50]).

**Fig. 3:**
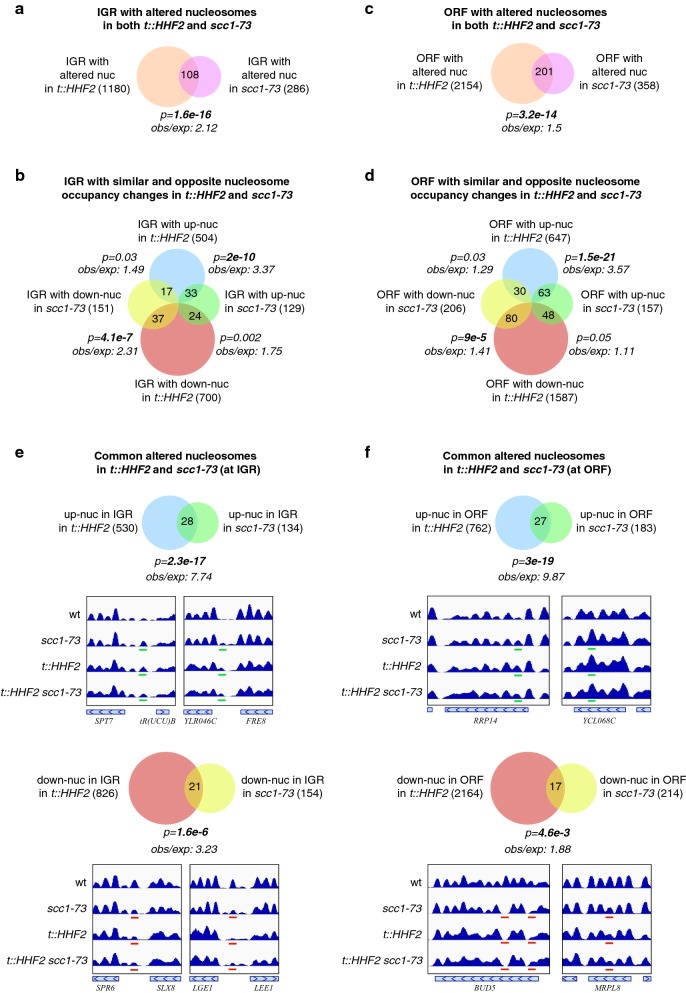
A lack of cohesin activity and histone depletion lead to similar occupancy changes in a subset of nucleosomes. **a**, **c** Overlap between IGR (**a**) or ORF (**c**) with altered nucleosomes in *t::HHF2* or *scc1*-*73* cells. **b**, **d** Overlap between IGR (**b**) or ORF (**d**) with either up- (increased occupancy) or down-nucleosomes (decreased occupancy) in *t::HHF2* and *scc1*-*73*. **e**, **f** Overlap between up- (increased occupancy) or down-nucleosomes (decreased occupancy) in IGR (**e**) and ORF (**f**) in *t::HHF2* and *scc1*-*73* cells. Representative examples are shown; up- and down-nucleosomes are marked in green and red, respectively. The number of genomic regions (**a**–**d**) or nucleosomes (**e**, **f**) in each case is indicated in parentheses. The probability of producing the given overlap if their distributions were random was generated using a hypergeometric test. The rate between the observed and expected frequencies is also shown

A comparative analysis showed that both the profile of genomic regions with altered nucleosomes and the class of changes in nucleosome occupancy in these genomic regions coincided in many cases in *scc1*-*73* and *t::HHF2* (Additional file 1: Table S1 and Additional file 2: Table S2). This observation prompted us to study if nucleosomes with altered occupancy lie at the same genomic regions in both mutants. Statistical analyses of IGRs with altered nucleosomes showed a highly significant number of common elements (Fig. 3a). Importantly, overlapping IGR shared nucleosomes with the same change—either increased or decreased—in occupancy (Fig. 3b). Similar results were obtained from the analysis of ORF with altered nucleosomes (Fig. 3c, d). A deeper analysis showed that this overlap was due to the fact that *t::HHF2* and *scc1*-*73* share a highly significant number of altered nucleosomes with the same change (Fig. 3e, f), especially in nucleosomes with increased occupancy (8 to 10 times more than expected). Moreover, manual inspection of these nucleosomes showed that the change in the double-mutant *t::HHF2 scc1*-*73* was similar to that displayed by the single mutants for 92% of nucleosomes (see representative examples, Fig. 3e, f). Altogether, these results suggest that part of the chromatin changes induced by defective nucleosome assembly and cohesin inhibition occurs through the same mechanism (~ 17% of *scc1*-*73*-altered nucleosomes).

### Mutant *scc1*-*73* cells do not display defects in the deposition of newly synthesized histones during DNA replication

The epistatic effect of *scc1*-*73* and *t::HHF2* on the integrity of a subset of nucleosomes might reflect a role for cohesins in histone deposition. Indeed, our RNA-seq analysis showed that the *scc1*-*73* mutant was specifically affected for expression of histones H4 and Htz1, as well as of histone chaperones Nap1 and Chz1 (Additional file 3: Table S3). To determine whether cohesins regulate histone deposition, we analyzed the incorporation into chromatin of newly synthesized histones at replicating DNA regions by following acetylated H3 at lysine K56 (H3K56ac) [51]. Although only a few nucleosomes were affected in *scc1*-*73* cells, we speculated that recycling parental histones could mask a major defect in the deposition of newly synthesized histones.

**Fig. 4:**
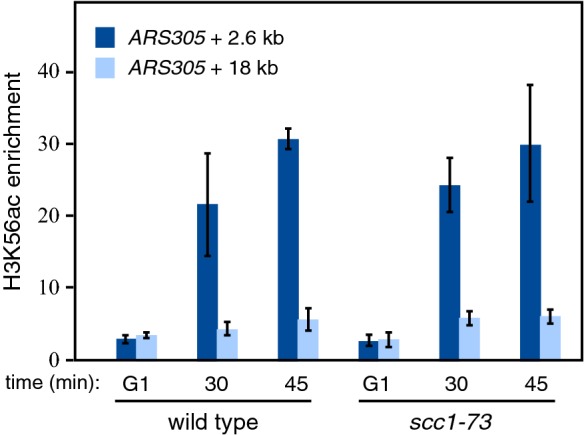
Cohesins are not involved in the deposition of newly synthesized histones. ChIP analysis of the incorporation of newly synthesized histone H3 (acetylated in lysine 56) in wild-type and *scc1*-*73* cells that have been synchronized in G1 and released into S phase in the presence of 200 mM HU for 45 min. H3K56c enrichment both at the proximity of *ARS305* and at an unreplicated region (2.6 and 18 kb from the origin, respectively) was calculated as the amount of DNA immunoprecipitated with an antibody against H3K56ac relative to that obtained with antibody against total histone H3. The average and range from two independent experiments are shown. IgG-treated cells were used as an internal control to confirm the specific enrichment at each region

H3K56ac accumulation at replicating DNA regions from origin *ARS305* was analyzed in cells synchronized in G1 and released into S phase in the presence of hydroxyurea, which reduces the pool of dNTPs and causes replication forks to be stalled in the proximity of the origin [52]. As expected, H3K56Ac accumulated around the origin (2.6 kb from *ARS305*) but not at an unreplicated DNA region (18 Kb from *ARS305*). Importantly, the absence of cohesin activity in *scc1*-*73* cells did not affect H3K56ac incorporation (Fig. 4), suggesting that cohesins do not have a major role in depositing new histones during DNA replication.

### Histone depletion has modest effects on the accumulation and distribution of cohesin peaks

**Fig. 5:**
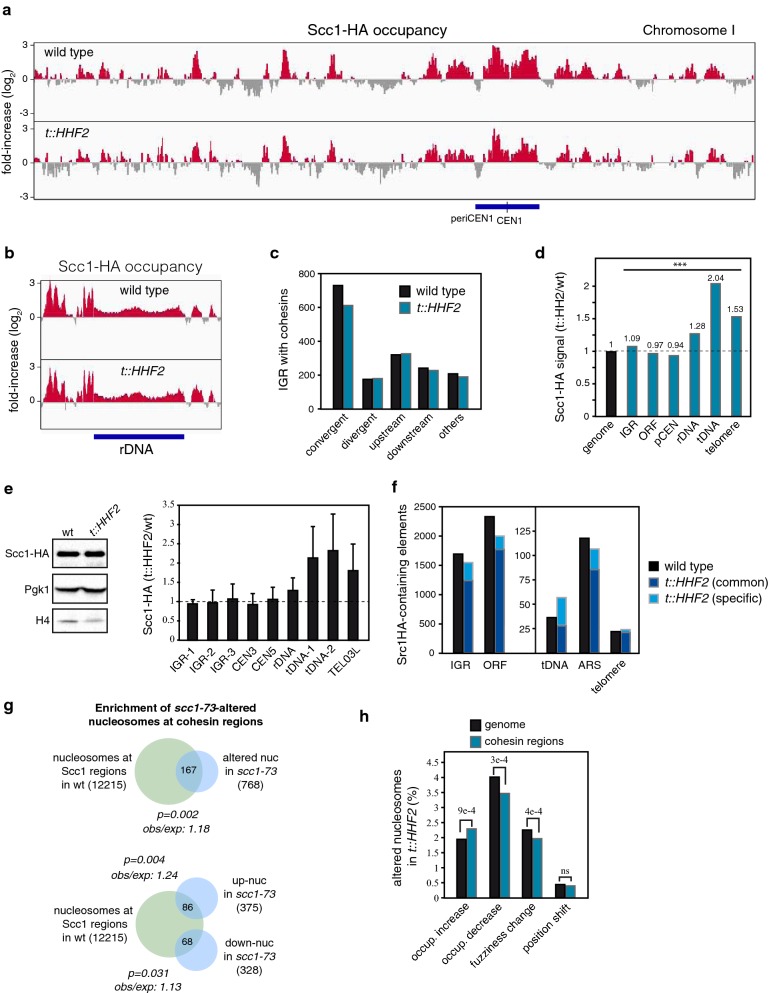
Histone depletion has a moderate effect on cohesin binding and distribution. **a** Cohesin distribution at chromosome I in wild-type and *t::HHF2* cells synchronized in G1 and released into fresh medium until G2-metaphase, as determined by ChIP-on-chip analysis against Scc1-HA. **b** Cohesin distribution at the ribosomal DNA locus in wild-type and *t::HHF2* cells. **c** Number of IGRs classified according to the orientation of the flanking genes that overlap with cohesins (by at least 1 bp) in wild-type and *t::HHF2* cells. **d** Relative amount of cohesins in *t::HHF2* cells relative to wild-type cells at the indicated genomic regions. The total amount of cohesins at each region was calculated considering the sum of positive signals (relative to the untagged strain) with a *p *< 0.05. The ratio between the mutant and the wild-type at each region was normalized to that obtained for the whole genome, which was taken as 1. The proportion of cohesins between mutant and wild-type cells at each genomic region relative to the genome average was statistically different according to a two-tailed Chi-square test (*p *< 0.001). **e** Cohesin enrichment in *t::HHF2* cells relative to wild-type cells at different genomic regions, as determined by ChIP and qPCR analyses against HA-Scc1 in cells grown as in **a**. IGR and tRNA genes are indicated in Methods section. Cohesin enrichment was calculated as the ratio between immunoprecipitated DNA and input in *t::HHF2* cells relative to the same value in the wild-type cells. The average and SEM from 3 to 4 independent experiments are shown. An untagged strain was used as an internal control to confirm the specific enrichment at each region. The amount of Scc1 relative to Pgk1 is shown on the left. No significant differences were observed between wild-type and mutant cells from four independent measurements. **f** Number of IGRs, ORF, tRNA, ARS and telomeres that overlap with cohesins (by at least 1 bp) in wild-type and *t::HHF2* cells. **g** Probability that *scc1*-*73* alters cohesin-associated nucleosomes if *scc1*-*73*-altered nucleosomes were randomly distributed, as determined by a hypergeometric test. The rate between the observed and expected frequencies of common nucleosomes is also shown. The number of nucleosomes in each case is indicated in parentheses. **h** Comparison of the percentages of altered nucleosomes by histone depletion (showing occupancy, fuzziness and position shift) in the regions with cohesins (9596 nucleosomes) relative to the whole genome (66,278 nucleosomes) in *t::HHF2* cells. A hypergeometric test was used to determine the probability of obtaining the indicated percentages of altered nucleosomes at regions with cohesin if their distributions were random

To further understand the interplay between cohesins and chromatin structure we studied the importance of chromatin integrity in cohesin binding and distribution. For this, cells were synchronized in G1 and released into fresh medium until G2/M under conditions of histone depletion at 30 °C, and the association of Scc1 was followed by a high-density ChIP-on-chip analysis (5-bp resolution). This analysis showed an overall similarity of cohesin peaks in *t::HHF2* and wild-type cells (Fig. 5a, b and Additional file 9: Fig. S2a), with cohesins preferentially bound to the pericentromeric chromatin, rDNA, tRNA genes and the IGR of convergently transcribed genes (Fig. 5a–c) [11–13]. However, the amount of cohesins at rDNA, tRNA genes and telomeres relative to the genome average was higher in histone-depleted cells than in wild-type cells (Fig. 5d and Additional file 5: Table S5). As the ChIP-on-chip analysis was not normalized with an internal control, no comparison between absolute amounts of signal between wild-type and mutant could be made; thus, we performed quantitative PCR from ChIP samples to validate the changes observed in cohesin binding. The absolute amount of cohesins at the major sites of cohesin binding (IGR and centromeres) was not affected, whereas higher levels of cohesins were detected at rDNA, tRNA genes and telomeres (Fig. 5e). While telomeres and rDNA were enriched in nucleosomes with increased and decreased occupancy, respectively, the percentage of altered nucleosomes at tDNA genes was below the whole genome average (Additional file 1: Table S1 and Additional file 2: Table S2).

Apart from these region-specific changes, the genome-wide analysis showed a loss of cohesins in 465 IGRs, and a gain of cohesin peaks in 315 IGRs that did not accumulate cohesins in the wild-type cells. Similar redistributions of cohesins were observed in ORF, tDNA genes, ARS and telomeres (Fig. 5f). This redistribution was not associated with preferential alterations in chromatin relative to non-affected regions, except for a slight increase in nucleosomes with higher occupancy (Additional file 9: Fig. S2b). Likewise, there was no significant overlap between IGR that had lost cohesins in *t::HHF2* and the IGR with altered nucleosomes in both *t::HHF2* and *scc1*-*73* (Additional file 9: Fig. S2b), suggesting that shared nucleosome alterations are not due to the loss of cohesin peaks in histone-depleted cells. In sum, defective chromatin integrity in histone-depleted cells has a moderate impact on cohesin binding, which is mostly evidenced by a redistribution of a subset of cohesin peaks and a higher accumulation at specific genomic regions.

### Cohesins have a slight contribution to maintaining chromatin integrity at cohesin-enriched regions

The MNase-seq analysis showed that cohesins contribute to the formation of a correct chromatin structure at specific genomic regions. To gain insight into the effects of cohesins on chromatin structure, we analyzed the position of altered nucleosomes in *scc1*-*73* cells relative to the distribution of cohesins in wild-type cells. We found that nucleosomes that overlap with cohesin regions have a slight but significant higher probability of being altered in *scc1*-*73* cells (Fig. 5g). We observed the same effect after analyzing genomic regions with altered nucleosomes in *scc1*-*73* cells; namely, altered nucleosomes were preferentially at IGR and ORF that contained cohesin peaks in wild-type cells (Additional file 6: Table S6). Thus, the loss of chromatin integrity upon cohesin inactivation is slightly more severe in cohesin-enriched regions.

Finally, a cohesin distribution analysis allowed us to address how cohesins influence chromatin disruption by histone depletion. A comparative analysis with the whole genome showed that regions with cohesins display higher levels of nucleosomes with increased occupancy and lower levels of nucleosomes with decreased occupancy and increased fuzziness (Fig. 5h), suggesting that cohesins prevent the loss of nucleosome positioning upon histone depletion.

### Upregulated genes in *scc1*-*73* cells are enriched in cohesins

As the elimination of cohesin activity during S phase affects gene expression, we asked whether this misregulation was associated with a direct binding of cohesins to regulated genes. Remarkably, upregulated, but not downregulated, genes in *scc1*-*73* cells displayed a highly significant enrichment in cohesin-binding genes (Table 2), suggesting that cohesin peaks have a repressor role.

## Discussion

### Cohesins contribute to structuring chromatin

Here we provide evidence that cohesins help to determine the primary chromatin structure of specific DNA regions. The absence of cohesin activity in the *scc1*-*73* mutant altered the occupancy of hundreds of nucleosomes. This effect was more pronounced in IGR, leading to both gain and loss of nucleosome occupancy. In addition, the occupancy of nucleosomes − 1 and + 1 at promoters was specifically increased on average in a transcription-independent (+ 1) and partially dependent (− 1) manner. These results suggest that cohesins have a subtle but significant effect on the architecture of yeast promoters. Formally, we cannot rule out that some of the chromatin changes in the *scc1*-*73* mutant stem from transcriptional defects, although the use of a conditional mutant reduces this possibility. Note that this does not mean that cohesins affect nucleosome positioning and/or transcription by acting directly on the altered nucleosomes and/or genes; our results show that a lack of cohesin activity affects the integrity of nucleosomes at both cohesin-associated and cohesin-free regions. Cohesins might help to shape cohesin-associated chromatin regions by either preventing histone deposition and/or facilitating nucleosome positioning, depending on additional chromatin determinants. Likewise, the effects of cohesin on nucleosomes at “cohesin-free” regions (as defined here and in other studies [11–13]) might be direct, as analyzing these regions with more sensitive methods reveals an accumulation of basal levels of cohesins [53]. Alternatively, nucleosome alterations at cohesin-free regions might result from cohesin activity at a distance, through topological constrains on adjacent chromatin fragments [54], as cohesins are major determinants of chromatin *cis*-loops from human to yeast [55–57]. Finally, chromatin defects might be indirectly generated by impairment of other cohesin-regulated processes. Similar arguments are valid for the effects of *scc1*-*73* in transcription.

Notably, a significant number of altered nucleosomes in *scc1*-*73* cells (~ 17%) were shared by *t::HHF2* and *t::HHF2 scc1*-*73* cells, suggesting that they occur through a common mechanism. We have discarded a major role for cohesins in the deposition of newly synthesized histones, even though it is still possible that the absence of cohesins affects recycling of parental histones, as sister chromatid entrapment by cohesin during replication and parental histone deposition are both associated with the replication fork [1, 58–61].

### Transcription regulation by cohesins

Studies in Drosophila and vertebrates using cohesin mutants have revealed both up- and downregulation of genes controlling development, proliferation and pluripotency [23, 62]. Our genome-wide transcription analysis showed a high number of misregulated genes in the *scc1*-*73* mutant (~ 15% with a > 1.5-fold change; ~ 4% with a > 2-fold change); these numbers are in the range of those obtained by the absence of the chromatin remodelers SWR or RSC, which have genome-wide transcriptional roles (7% and 17% with > 1.5-fold change for *swr1∆* and *sth1*-*3*, respectively) [26, 46]. Indeed, a global gene expression analysis of 132 mutants of chromatin regulators with altered transcription profiles showed that the average percentage of genes with a significant change (fold change > 1.7) was ~ 2% and that the largest effect was 16% [63]. Therefore, our results support a major role for cohesins in transcription regulation in yeast.

**Fig. 6:**
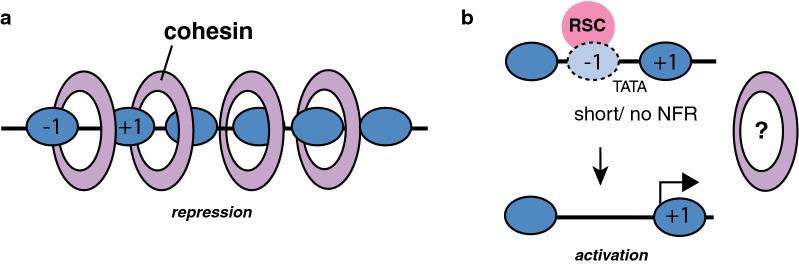
Hypothetical model for gene regulation by cohesins. **a** Upregulated genes in *scc1*-*73* cells are preferentially in cohesin-enriched regions. Cohesin accumulation might recruit transcription repressors, generate a locally condensed chromatin or hamper the recruitment and/or movement of the transcription machinery. **b** Downregulated genes in *scc1*-*73* cells are enriched in promoters that have a short or no NFR and a fragile − 1 nucleosome. These promoters require the RSC complex together with additional transcriptional factors to generate a NFR and to activate transcription. Cohesins might collaborate with RSC and other chromatin remodeling factors to remove the nucleosome and/or maintain the NFR

We do not rule out that cohesins play a role in transcription by promoting DNA looping, as proposed for vertebrates [64]. However, transcription analyses in the *scc1*-*73* mutant relative to cohesin accumulation and chromatin structure, together with the effects of cohesins on nucleosome occupancy at promoters, suggest additional (but not mutually exclusive) mechanisms of transcription regulation by cohesins. First (and in sharp contrast to *scc1*-*73* downregulated genes, which show a random distribution), upregulated genes in *scc1*-*73* cells mainly are in cohesin-enriched regions. The nucleosome profile of upregulated genes in *scc1*-*73* cells was not associated with specific chromatin changes. One possibility is that cohesins facilitate transcription repression by compacting chromatin without altering nucleosome occupancy. Alternatively, cohesin accumulation might inhibit transcription by specifically recruiting repression factors and/or hampering the binding and/or movement of the transcription machinery (Fig. 6a). In any case, it is particularly interesting that upregulated genes in *scc1*-*73* cells are enriched in genes that require the NuA4 complex for activation, as it opens the possibility that histone acetylation is required to counteract a putative repressor role by cohesins.

On the other hand, downregulated genes in *scc1*-*73* cells were characterized by promoters containing TATA and regulated by the Mediator, SAGA and SWR complexes. This suggests that, regardless of the putative mechanisms of action, chromatin dynamics plays a major function in the activation of these genes. Critically, the role of cohesins in transcription activation seems to be associated with a specific chromatin structure at promoters. Thus, downregulated genes were enriched in promoters with short or no NFR and a fragile nucleosome at the position of nucleosome − 1. The proposal of this fragile nucleosome [39] has been challenged by data supporting the presence of non-histone protein complexes [65]. However, recent analysis revealed the presence of the RSC complex and global transcription factors bound to a partially unwrapped nucleosome intermediate [66]. In addition to removing this nucleosome, these factors seem to shift apart the flanking nucleosomes, exposing the TBP-binding site, which facilitates transcription (Fig. 6b) [39, 40, 66, 67]. In accordance with a putative role for cohesins in the regulation of genes with FN promoters, downregulated genes in *scc1*-*73* cells were enriched in RSC/nucleosome complexes at their promoters. Interestingly, the occupancy of nucleosome − 1 was particularly increased in *scc1*-*73*-downregulated genes. Taking into account these associations, one possibility is that cohesins help RSC to remove the nucleosome − 1 and/or to maintain the NFR (Fig. 6b). In this light, it is worth noting that RSC facilitates the recruitment to promoters of the cohesin loader Scc2/Scc4, which in turn helps RSC to maintain the NFR [26]. Moreover, RSC interacts directly with both cohesin and Scc2/Scc4 and is required for cohesin loading [27, 28]. However, the Scc2/Scc4 complex was not preferentially associated with downregulated genes in *scc1*-*73* cells, suggesting that cohesins operate in transcription independently of Scc2/Scc4. Alternatively, transient and dynamics interactions between RSC, Scc2/Scc4 and cohesins may be required for chromatin remodeling and transcription regulation. Further genomic and gene-specific time-course molecular analyses will be required to validate these hypothetical models, and to determine if the role of cohesins in transcription in *S. cerevisiae* is also related to its DNA looping activity.

The increase in occupancy at nucleosome − 1 is more severe (but not specific) for *scc1*-*73*-downregulated genes. Further, the lack of cohesin activity causes a global effect on the occupancy of nucleosome + 1, a nucleosome that plays a key role in the regulation of transcription. Nascent mRNA studies will be required to establish if the effects of *scc1*-*73* on nucleosomes − 1 and + 1 are associated with a more general role of cohesins in transcription regulation.

### Chromatin integrity plays a moderate role in global cohesin binding and distribution

Until now, whether (and how) chromatin structure contributes to cohesin binding and distribution has been controversial. Recently, Uhlmann and colleagues provided strong in vivo and in vitro evidence for an inhibitory role of nucleosomes in cohesin loading [28]. Here, we show that the amount of cohesins was not increased in most genomic regions after histone depletion and that there was not a preferential loss of nucleosome occupancy in those regions in which cohesins accumulated relative to the wild type. Therefore, the amount of loaded cohesins is not limited by chromatin under wild-type conditions. Remarkably, the general pattern of cohesin peaks as well as the preferential regions of cohesin binding remained mostly unaffected despite a severe loss of chromatin integrity. Therefore, the global pattern of cohesins throughout the genome does not seem to require a precise chromatin organization. Furthermore, the number of cohesin peaks did not augment despite a global reduction in nucleosome density, suggesting that NFRs per se are not preferential sites for cohesin accumulation. Although these results do not discard the requirement of specific histone interactions for cohesin binding and/or distribution, they point to DNA-associated specific features (e.g., A/T-rich DNA sequences, positively supercoiled DNA) [14, 68] and DNA metabolic processes (e.g., transcription, DNA replication) [11–14, 59, 69, 70], as major determinants for cohesin distribution in vivo. Indeed, the changes in cohesin distribution and accumulation after histone depletion might be due to defects in transcription and/or replication fork stability, as these processes are severely impaired in histone-depleted cells [71, 72].

## Conclusions

In summary, our study has explored some of the connections between cohesin activity, chromatin integrity and transcription. We show that the binding and distribution of the cohesin peaks do not require a precise nucleosome organization, whereas chromatin integrity relies on cohesin activity at some regions, including promoters. These observations, together with the association of the cohesin-regulated genes with specific promoter chromatin architecture, suggest a role for chromatin dynamics in the regulation of transcription by cohesins. Remarkably, OPN genes (and more specifically SAGA-dominated genes) are characterized by high transcriptional plasticity [37, 43], which is a requirement for genes that control development, proliferation and pluripotency. In humans, the genes with the highest nucleosome occupancy at the TSS-proximal region (OPN-like genes) display high transcriptional plasticity [37]. It will therefore be important to study the chromatin structure of cohesin-regulated promoters in humans, as well as the impact that cohesins may have on the more complex human chromatin structure. This may be particularly relevant to understanding cohesinopathies, such as Cornelia de Lange syndrome and Roberts syndrome, which are associated with cohesin mutants that are proficient in cohesion but defective in gene expression [73].

## Methods

### Yeast strains, plasmids and growth conditions

Yeast strains used in this study are listed in Additional file 7: Table S7a. Tagged strains and deletion mutants were constructed by a PCR-based strategy [74]. For G1 synchronization, cells were grown in supplemented minimal medium (SMM) to mid-log-phase and α factor was added twice at 90-min intervals at 0.5 μg/ml, except for *t::HHF2* strains, which were treated with 1 μg/ml. Cells were then washed three times and released into fresh SMM with 50 μg/ml pronase, except for cells released in the presence of nocodazole, which were released in rich medium (YPAD). To induce nucleosome depletion, *t::HHF2* cells growing in the presence of 5 μg/ml doxycycline were shifted to 0.25 μg/ml during G1 synchronization and release. Cell cycle progression was followed by flow cytometry, budding index and DAPI staining.

### Western blot

Protein extracts were obtained from cell cultures treated with 0.1 M sodium azide to stop cell growth. Briefly, 25 ml cultures were incubated in ice-cold 0.1 Tris–HCl pH 9.4, 10 mM DTT solution for 15 min, collected by centrifugation, washed with 20 mM HEPES pH 7.4, 1.2 M sorbitol, 1 × cocktail inhibitor (Roche), and incubated in the same solution with 0.21 mg of zymolyase 20T for 1 h at 30 °C. Spheroplasts were then washed twice with 20 mM Tris–HCl pH 7.5, 20 mM KCl, 1 M sorbitol, 1 × cocktail inhibitor, 0.1 μM spermidine and 0.25 μM spermine and lysed with cold 20 mM Tris–HCl pH 7.5, 20 mM KCl, 0.4 M sorbitol, 1% Triton, 1 × cocktail inhibitor, 0.1 μM spermidine and 0.25 μM spermine solution for 5 min. Lysed extracts were mixed with Laemmli buffer, boiled and run on an SDS–polyacrylamide gel. Proteins were transferred to a nitrocellulose membrane (Hybond-ECL) that was blocked in PBS-T milk 5% and incubated with primary antibodies against HA (rat monoclonal 3F10, Roche), Pgk1 (mouse polyclonal 22C5D8, Invitrogen), or histone H4 (rabbit polyclonal ab10158, Abcam). Proteins were detected using a peroxidase-conjugated goat anti-mouse or anti-rabbit IgG (both from Bio-Rad) secondary antibody and the ChemiDoc Gel Imaging System.

### Chromatin analysis by MNaseI digestion and indirect end labeling

G2/M cells were fixed for 15 min with 1% formaldehyde. Glycine was added to quench the reaction at a final concentration of 125 mM. Cells were sedimented, washed twice with cold TBS and stored at − 80 °C until use. Extracts for MNase digestion were re-suspended in 1 M sorbitol and digested 1 h with 4.5 mg of zymolyase 20T (AmsBio 120491-1). Samples were washed first with 1 M sorbitol and then with 1 M sorbitol 0.1 mM PMSF, suspended gently in solution II (20 mM Tris–HCl, 2 mM EDTA, 0.15 M NaCl, 0.1 mM PMSF, 0.2% Triton), and treated 30 min with different concentrations of MNase (SIGMA N3755). The reaction was then stopped by adding 0.4% SDS, 8.5 mM EDTA. To revert cross-linking, samples were incubated for 90 min at 37 °C with proteinase K and then overnight at 65 °C. DNA was extracted from samples using a standard phenol–chloroform extraction, treated with RNase A and loaded in a 1% agarose gel to check MNase digestion. MNase digestions used for indirect end labeling were incubated with the indicated restriction enzymes, resolved in 1.5% agarose gels, blotted onto a HybondTM-XL membrane and probed with ~ 200 to 250 bp ^32^P-labeled specific PCR fragments. These fragments were always located close to one of the two ends of the fragment analyzed. Oligonucleotides for PCR amplification are listed in Additional file 7: Table S7b. Signals were acquired in a Fuji FLA5100 with the Image Gauge analysis program.

### Chromatin analysis by MNase-seq

MNaseI-digested DNA samples from two biological replicates for each yeast strain were obtained as previously indicated for indirect end labeling. MNase-digested samples enriched in mononucleosomes were loaded in a 1% agarose gel, and the DNA corresponding to mononucleosomes was purified with a DNA purification kit (Qiagen). The DNA size and quality were confirmed by an electropherogram analysis (2100 Bioanalyzer™) (Additional file 10: Fig. S3). Library construction and sequencing was performed at Genomics Core Facility of CABIMER. DNA libraries were prepared from 100 ng mononucleosome DNA using the Ion Plus Fragment Library Kit (Thermo Fisher), and the size distribution and molarity of each library were analyzed with the Agilent™ DNA High Sensitivity Kit (Agilent 2100 Bioanalyzer). DNA libraries were sequenced on the Ion Torrent™ Personal Genome Machine™ (PGM), and raw data were processed for base calling, filtering and trimming to generate the FASTQ files using the Torrent Suite™ Software. Sequence reads were mapped to *S. cerevisiae* genome sacCer3 by BowTie2 [75], and potential PCR duplicates were removed by SAM Tools on the Galaxy platform (usegalaxy.org) [76]. The peak-calling algorithm *Dpos* function (DANPOS 2.2.0) [35, 77] was used for nucleosome occupancy maps and comparative analyses using default parameters. Dynamic nucleosomes were selected using a point_diff_log10pval > -15, and they were classified into three categories: position shift (range setting between 50 and 90 bp), fuzziness (fuzziness_diff_log10pval > -15) and occupancy changes (smt_diff_log10pval > -15). Average nucleosome distance, occupancy and fuzziness were analyzed and plotted with the *stat* function (DANPOS). Average nucleosome occupancy patterns flanking transcription start sites (TSS) were plotted in either average density or heat maps using *Profiles* function (DANPOS) or DeepTools (usegalaxy.org), respectively [78].

### Chromatin immunoprecipitation (ChIP)

Cells in the indicated conditions were fixed for 15 min with 1% formaldehyde. Glycine was added to quench the reaction at a final concentration of 125 mM. Cells were sedimented, washed twice with cold TBS and stored at − 80 °C until use. ChIP samples were obtained by breaking cells with a homogenizer (Multibeads shocker, Yasui Kikai) for 1 h at 2500 rpm (30-s on/30-s off intervals) in lysis buffer (50 mM HEPES, 140 mM NaCl, 1 mM EDTA, 1% Triton, 0.1% sodium deoxycholate, 1 mM PMSF) supplemented with protease cocktail inhibitors (Roche). Supernatant was transferred to new tubes by soft centrifugation piercing at the bottom of the tube with a G25 needle, and chromatin was further concentrated by centrifugation. Chromatin was sheared via sonication to a size between 200 and 600 bp using a sonicator (Branson Digital Sonifier, Branson Ultrasonics). About 10 µl of supernatant was kept on ice and used as the input DNA control; the rest was incubated overnight with 1 µg of antibody at 4 °C. Antibodies used include IgG (A4416, Sigma), anti-HA (3F10, Roche), anti-H3K56Ac (39281, Active Motif) and anti-H3 (AB1791, Abcam). All samples were immunoprecipitated using magnetic Protein G Dynabeads (Invitrogen; 10003D) for 90 min. After immunoprecipitation, samples were washed twice with 1 ml of the following solutions: lysis buffer, lysis buffer plus 0.5 M NaCl, wash buffer (0.25 M LiCl, 10 mM Tris–HCl, 1 mM EDTA, 0.5% NP-40, 0.5% sodium deoxycholate) and TE 1 × . Samples were then eluted from magnetic beads with a 1% SDS TE solution, incubated overnight at 65 °C to reverse cross-linking, treated with 0.15 mg of proteinase K and extracted using a standard phenol/chloroform DNA purification. ChIP data were obtained by real-time qPCR using SYBR Green Premix Ex Taq (Takara). In Fig. 5e, IGRs 1–3 contained the promoters of *ERR2*, *IWR1* and *GAT3*, and tDNA 1 and 2 are tP(UGG)N1 and tK(CUU)E1 (see Additional file 1: Fig. S7b for primers).

### Cohesin binding by ChIP-on-chip

High-resolution (5-bp) ChIP-on-chip analyses were performed with two biological samples for wild type and *t::HHF2*. In each case, input (I) and immunoprecipitated (IP) DNA with antibody against HA from HA-Scc1 and untagged cells grown under the indicated conditions were obtained by ChIP (as described above) and then additionally purified through a DNA purification column (Qiagen). DNA was amplified by random priming using a GenomePlex^®^ Whole Genome Amplification (WGA) kit and cleaned with GenElute™ PCR Clean-Up Kit (Sigma-Aldrich). A total of 7.5 μg of amplified DNA was fragmented, labeled and hybridized with the GeneChip *S. cerevisiae* Tiling 1.0R array (Affymetrix Inc.) following the manufacturer’s procedure (25-bp probes shifted every 5 bp). ChIP-on-chip data were analyzed using the Tiling Array Suite 1.1.02 (TAS) software from Affymetrix. TAS produces the signal (log2 ratio IP/I) and the *p* value intensity files per probe position (150 bp bandwidth around the inspected probe using quantile normalization plus scaling). Positive enrichment intervals were filtered as the regions with *p* value < 0.05 and a positive signal [(IP/I) − (IP/I)untag], considering a maximum gap of 250 bp and a minimum run/length of 30 bp.

### Transcription analysis by RNA-seq

RNA was extracted using a standard hot acid phenol extraction protocol [79] and purified with the RNeasy Mini kit (Qiagen). RNA samples were analyzed for quality with the RNA 6000 Nano assay on a 2100 Bioanalyzer (Agilent Technologies) and quantified with the Qubit™ RNA HS Assay (Thermo Fisher Scientific™). RNA libraries from two independent biological replicates for wild type and *scc1*-*73* were constructed and sequenced at the Genomics Core Facility of CABIMER. For this, strand-specific total RNA-seq libraries from 100 ng RNA samples were prepared using the TruSeq^®^ Stranded mRNA Library prep kit (Illumina). Indexed libraries were pooled and sequenced on Illumina NextSeq 500 using paired-end chemistry with 75 bp read length to a depth of approximately about 37 million reads per library. Raw reads were filtered and trimmed with FASTQ toolkit 1.0.0 and assessed using FastQC 1.0.0 by BaseSpace Sequence Hub Illumina website. FASTQ data were uploaded to the Galaxy web platform for further analyses [76]. Briefly, reads were aligned to the *S. cerevisiae* genome version sacCer3 using the HISAT2 aligner [80] and filtered for high-quality mapping (MapQuality ≥ 30) using BAM tools [81]. Differential expression analyses including fold change and statistical significance of gene expression profiles were performed using the Cuffdiff program [82] and the reference annotation sacCer3.gtf (downloaded from UCSC). Gene ontology analyses were performed with the DAVID Bioinformatics resources [83].

### Genome-wide data

Nucleosome profiles and cohesin peaks along the genome were visualized using the Integrative Genomics Viewer (IGV) [84]. All data are MIAME compliant. Raw data have been deposited at the MIAME-compliant Gene Expression Omnibus (GEO) database at the National Center for Biotechnology Information (http://www.ncbi.nlm.nih.gov/geo/) and are accessible through the accession numbers GSE121067 (MNaseI-seq), GSE121004 (ChIP-on-chip) and GSE125258 (RNA-seq).

### Comparative analyses of genomic elements

Analyses of the genome distribution and overlapping of dynamic nucleosomes, cohesin peaks and gene elements were performed with the tools of the Galaxy web platform (usegalaxy.org) [76]. Other statistical analyses were performed with the GraphPad Prism software.

## Additional files

**Additional file 1: Table S1**. Comparison of the number and percentage of nucleosomes altered after histone depletion (in *t::HHF2* cells) or cohesin inactivation (in *scc1*-*73* cells) in the indicated genomic regions relative to the whole genome. A hypergeometric test was used to determine the probability to obtain the indicated percentages of altered nucleosomes at the indicated genomic regions if their distribution along the genome were random. Significantly higher (green) and lower (red) percentages are highlighted. *rDNA is analyzed as a single copy.

**Additional file 2: Table S2**. Comparison of the number and percentage of nucleosomes altered after histone depletion (in *t::HHF2* cells) or cohesin inactivation (in *scc1*-*73* cells) in the indicated genomic regions relative to the whole genome. A hypergeometric test was used to determine the probability of obtaining the indicated percentages of altered nucleosomes at the indicated genomic regions if their distribution along the genome were random. Significantly higher (green) and lower (red) percentages are highlighted. *rDNA, analyzed as a single copy.

**Additional file 3: Table S3**. Misregulated genes in *ssc1*-*73*. Lists of up- and downregulated genes are shown.

**Additional file 4: Table S4** List of misregulated genes from additional studies used for comparative analyses in Table 2.

**Additional file 5: Table S5**. Cohesin distribution in wild-type and histone-depleted cells at the indicated genomic regions. A peak of Scc1 is defined as a DNA fragment with continuous Scc1 signals that are both positive (relative to the untagged strain) and with a *p *< 0.05. A peak signal was calculated as the sum of these positive signals. Genomic regions with Scc1 and peaks of Scc1 at a particular genomic region are defined by at least 1 bp overlapping.

**Additional file 6: Table S6.** The effect of cohesin inactivation on chromatin structure is more severe in cohesin-associated regions. The probability that *scc1*-*73* alters nucleosomes in Scc1-binding IGR or Scc1-binding ORF if the altered genomic regions in *scc1*-*73* cells were randomly distributed was determined by a hypergeometric test. The rate between the observed and expected frequencies of common genomic regions is also shown. The number of regions in each case is indicated in parenthesis.

**Additional file 7: Table S7**. *Saccharomyces cerevisiae* strains **(a)** and oligos **(b)** used in this study.

**Additional file 8: Fig. S1**. Effect of cohesin inactivation in *scc1*-*73* cells on the chromatin structure of three different loci of histone-depleted cells. Nucleosome positioning analyses are shown for the indicated loci after MNase I digestion and indirect-end labeling of the indicated strains synchronized in G1 and released into fresh medium for 1 h at 37 °C until G2/M.

**Additional file 9: Fig. S2**. Effect of histone depletion on cohesin binding and distribution. **a** Cohesin distribution at different regions of chromosomes II and V in wild-type and *t::HHF2* cells that have been synchronized in G1 and released into fresh medium until G2/M, as determined by ChIP-on-chip analysis against HA-Scc1. **b** Probability that IGR that had either lost or gained cohesins after histone depletion would overlap with IGR with altered nucleosomes in *t::HHF2* cells or in both *t::HHF2* and *scc1*-*73* cells if they were randomly distributed, as determined by a hypergeometric test. The rate between the observed and expected frequencies of common IGR is also shown. The number of IGR in each case is indicated in parenthesis. nd, not determined.

**Additional file 10: Fig. S3**. Preparation and analysis of the nucleosomal DNA used for MNaseI-seq. **a** Generation of mononucleosomes in the indicated strains after partial digestions with MNase I. The DNA purified for DNA-seq is marked in red. **b, c** Electrophoretic (b) and electropherogram (c) analyses of the purified nucleosomal DNA.

## Data Availability

Genome-wide data have been deposited at the MIAME-compliant Gene Expression Omnibus (GEO). The remaining data and material are available from the corresponding author on request.

## Abbreviations

ARS: autonomous replication sequence
DANPOS: dynamic analysis of nucleosome position and occupancy by sequencing
DPN: depleted-proximal promoter
FN: fragile nucleosome
IGR: intergenic region
NFR: nucleosome-free region
OPN: occupied-proximal promoter
ORF: open reading frame
rDNA: ribosomal DNA
SN: stable nucleosome
tDNA: transfer DNA
TSS: transcription start site
