# Supplementary material for: Crosstalk between chromatin structure, cohesin activity and transcription
Source: Epigenetics Chromatin. 2019 Jul 22;12:47. doi: 10.1186/s13072-019-0293-6 (PMC6647288; doi:10.1186/s13072-019-0293-6)
Supplement: Supplementary file 1 — Additional file 1: Table S1. Comparison of the number and percentage of nucleosomes altered after histone depletion (in t::HHF2 cells) or cohesin inactivation (in scc1-73 cells) in the indicated genomic regions relative to the whole genome. A hypergeometric test was used to determine the probability to obtain the indicated percentages of altered nucleosomes at the indicated genomic regions if their distribution along the genome were random. Significantly higher (green) and lower (red) percentages are highlighted. *rDNA is analyzed as a single copy. [file 13072_2019_293_MOESM1_ESM.pdf]

**Genomic region**	***Nucles.***	***t::HHF2***		***scc1-73***	
		Altered nucleosomes (%)	***p-value***	***Altered nucleosomes (%)***	***p-value***
**genome**	66279	4431 (6.69)		768 (1.16)	
**IGR**	19557	**1478 (7.56)**	**8.6e-10**	**317 (1.62)**	**8.1e-13**
**ORF**	52047	**3298 (6.34)**	**1.9e-12**	**437 (0.84)**	**8.1e-42**
**Pericentric chromatin**	1726	125 (7.24)	0.024	15 (0.87)	0.051
**rDNA***	106	8 (7.55)	0.14	1 (0.94)	0.36
**tDNA**	285	**11 (3.86)**	**0.014**	**8 (2.8)**	**0.012**
**Telomeres**	784	**83 (10.59)**	**1.1e-5**	**20 (2.55)**	**6.0e-4**

Table S1. Comparison of the number and percentage of nucleosomes altered after histone depletion (in *t::HHF2* cells) or cohesin inactivation (in *scc1-73* cells) in the indicated genomic regions relative to the whole genome. A hypergeometric test was used to determine the probability to obtain the indicated percentages of altered nucleosomes at the indicated genomic regions if their distribution along the genome were random. Significantly higher (green) and lower (red) percentages are highlighted. *rDNA is analyzed as a single copy.
