# Supplementary material for: Crosstalk between chromatin structure, cohesin activity and transcription
Source: Epigenetics Chromatin. 2019 Jul 22;12:47. doi: 10.1186/s13072-019-0293-6 (PMC6647288; doi:10.1186/s13072-019-0293-6)
Supplement: Supplementary file 2 — Additional file 2: Table S2. Comparison of the number and percentage of nucleosomes altered after histone depletion (in t::HHF2 cells) or cohesin inactivation (in scc1-73 cells) in the indicated genomic regions relative to the whole genome. A hypergeometric test was used to determine the probability of obtaining the indicated percentages of altered nucleosomes at the indicated genomic regions if their distribution along the genome were random. Significantly higher (green) and lower (red) percentages are highlighted. *rDNA, analyzed as a single copy. [file 13072_2019_293_MOESM2_ESM.pdf]

Genomic region	*Nucles.*	***t::HHF2***						***scc1-73***					
		Fuzziness (%)	*p-val.*	*Occupancy decrease (%)*	*p-val.*	*Occupancy increase (%)*	*p-val.*	*Fuzziness (%)*	*p-val.*	*Occupancy decrease (%)*	*p-val.*	*Occupancy increase (%)*	*p-val.*
**genome**	66279	1516 (2.3)		2685 (4.05)		1304 (2)		77 (0.12)		375 (0.6)		328 (0.5)	
**IGR**	19557	**374 (1.9)**	**3e-6**	**826 (4.2)**	**6e-3**	**530 (2.7)**	**3e-18**	28 (0.14)	0.04	**154 (0.8)**	**1e-11**	**134 (0.7)**	**1.5e-3**
**ORF**	52047	**1331 (2.6)**	**2e-21**	**2164 (4.2)**	**5e-4**	**762 (1.5)**	**2e-61**	60 (0.12)	0.11	**214 (0.4)**	**1e-8**	**183 (0.35)**	**5e-37**
**Pericentromere**	1726	43 (2.5)	0.05	71 (4.1)	0.05	37 (2.1)	0.06	2 (0.12)	0.27	8 (0.46)	0.14	**5 (0.29)**	**0.04**
**rDNA***	106	0 (<1)	n.a.	**8 (7.5)**	**0.04**	0 (<0.9)	n.a.	0 (<1)	n.a.	0 (<0.9)	n.a.	1 (0.94)	0.33
**tDNA**	285	**2 (0.7)**	**0.03**	**3 (1.1)**	**2e-3**	7 (2.5)	0.13	0 (<0.4)	n.a.	0 (<0.4)	n.a.	**8 (2.8)**	**2e-4**
**Telomeres**	784	15 (1.9)	0.08	26 (3.3)	0.04	**51 (6.5)**	**1e-13**	2 (0.26)	0.17	2 (0.25)	0.15	**18 (2.3)**	**6e-7**
X-element	134	**15 (11)**	**4e-7**	0 (<0.8)	n.a.	**24 (17.9)**	**2e-16**	0 (<0.74)	n.a.	0 (<0.8)	n.a.	**13 (9.7)**	**1e-12**
Y-element	671	0 (<0.15)	n.a.	26 (3.9)	0.08	**35 (5.2)**	**2e-7**	2 (0.3)	0.14	2 (0.3)	0.2	**11 (1.6)**	**1e-3**
Telom. repeat	28	0 (<3.6)	n.a.	0 (<3.6)	n.a.	**14 (50%)**	**4e-17**	0 (<3.6)	n.a.	0 (<3.6)	n.a.	1 (3.6)	0.14

Table S2. Comparison of the number and percentage of nucleosomes altered after histone depletion (in *t::HHF2* cells) or cohesin inactivation (in *scc1-73* cells) in the indicated genomic regions relative to the whole genome. A hypergeometric test was used to determine the probability of obtaining the indicated percentages of altered nucleosomes at the indicated genomic regions if their distribution along the genome were random. Significantly higher (green) and lower (red) percentages are highlighted. *rDNA, analyzed as a single copy.
