# Supplementary material for: Crosstalk between chromatin structure, cohesin activity and transcription
Source: Epigenetics Chromatin. 2019 Jul 22;12:47. doi: 10.1186/s13072-019-0293-6 (PMC6647288; doi:10.1186/s13072-019-0293-6)
Supplement: Supplementary file 5 — Additional file 5: Table S5. Cohesin distribution in wild-type and histone-depleted cells at the indicated genomic regions. A peak of Scc1 is defined as a DNA fragment with continuous Scc1 signals that are both positive (relative to the untagged strain) and with a p < 0.05. A peak signal was calculated as the sum of these positive signals. Genomic regions with Scc1 and peaks of Scc1 at a particular genomic region are defined by at least 1 bp overlapping. [file 13072_2019_293_MOESM5_ESM.pdf]

	Wild type			*t::HHF2*			
**Genomic region**	**Regions with Scc1**	**Peaks of Scc1**	***peak signal (mean+SEM)***	**Regions with Scc1**	**Peaks of Scc1**	***peak signal (mean+SEM)***	**Total regions genome**
**Intergenic**	**1720**	**1277**	1,267+0,001	**1570**	**1216**	0,995+0,001	**6620**
**ORF**	**2363**	**1934**	1,224+0,001	**2034**	**1766**	0,970+0,001	**5775**
**tRNA_gene**	**38**	**37**	1,114+0,005	**58**	**57**	0,900+0,004	**275**
**telomere**	**23**	**47**	0,551+0,007	**24**	**44**	**0,700+0,009**	**32**

Table S5. Cohesin distribution in wild-type and histone-depleted cells at the indicated genomic regions. A peak of Scc1 is defined as a DNA fragment with continuous Scc1 signals that are both positive (relative to the untagged strain) and with a $p < 0.05$. A peak signal was calculated as the sum of these positive signals. Genomic regions with Scc1 and peaks of Scc1 at a particular genomic region are defined by at least 1 bp overlapping.
