# Supplementary material for: Crosstalk between chromatin structure, cohesin activity and transcription
Source: Epigenetics Chromatin. 2019 Jul 22;12:47. doi: 10.1186/s13072-019-0293-6 (PMC6647288; doi:10.1186/s13072-019-0293-6)
Supplement: Supplementary file 6 — Additional file 6: Table S6. The effect of cohesin inactivation on chromatin structure is more severe in cohesin-associated regions. The probability that scc1-73 alters nucleosomes in Scc1-binding IGR or Scc1-binding ORF if the altered genomic regions in scc1-73 cells were randomly distributed was determined by a hypergeometric test. The rate between the observed and expected frequencies of common genomic regions is also shown. The number of regions in each case is indicated in parenthesis. [file 13072_2019_293_MOESM6_ESM.pdf]

<table>
<tr><td></td><td></td><td>IGR with altered nuc. in scc1-73 (286)</td><td>IGR with up nuc. in scc1-73 (129)</td><td>IGR with down nuc. in scc1-73 (151)</td></tr>
<tr><td rowspan="2">Scc1-binding IGR (1720)</td><td>overlap.</td><td>84</td><td>32</td><td>50</td></tr>
<tr><td>obs/exp (p-val)</td><td>1.12 (0.02)</td><td>0.96 (0.08)</td><td>1.27 (0.01)</td></tr>
<tr><td colspan="2"></td><td>ORF with altered nuc. in scc1-73 (358)</td><td>ORF with up nuc. in scc1-73 (157)</td><td>ORF with down nuc. in scc1-73 (206)</td></tr>
<tr><td rowspan="2">Scc1-binding ORF (2363)</td><td>overlap.</td><td>167</td><td>80</td><td>91</td></tr>
<tr><td>obs/exp (p-val)</td><td>1.14 (0.03)</td><td>1.24 (0.002)</td><td>1.07 (0.03)</td></tr>
</table>

Table S6. The effect of cohesin inactivation on chromatin structure is more severe in cohesin-associated regions. The probability that *scc1-73* alters nucleosomes in Scc1-binding IGR or Scc1-binding ORF if the altered genomic regions in *scc1-73* cells were randomly distributed was determined by a hypergeometric test. The rate between the observed and expected frequencies of common genomic regions is also shown. The number of regions in each case is indicated in parenthesis.
