# Supplementary material for: Crosstalk between chromatin structure, cohesin activity and transcription
Source: Epigenetics Chromatin. 2019 Jul 22;12:47. doi: 10.1186/s13072-019-0293-6 (PMC6647288; doi:10.1186/s13072-019-0293-6)
Supplement: Supplementary file 7 — Additional file 7: Table S7. Saccharomyces cerevisiae strains (a) and oligos (b) used in this study. [file 13072_2019_293_MOESM7_ESM.pdf]

Table S7a. ***Saccharomyces cerevisiae*** **strains used in this study**

Strain	Genotype	Ref
YK402-2	*bar1Δ::HISG*	[1]
ws73-10B<br>ws73-4B	*scc1-73 bar1Δ::natMX4*	[2]<br>This work
wtH4-9b<br>wtH4-8D<br>DMY24<br>wtH4-sup	*hhf1Δ::hphMX4 hhf2Δ::kanMX4 (p413TAR tet:HHF2) bar1Δ::natMX4*	[2]<br>This work
wtH4s73-9B<br>wtH4s73-19B	*hhf1Δ::hphMX4 hhf2Δ::kanMX4 (p413TAR-tet:HHF2) bar1Δ::natMX4 scc1-73*	[2]<br>This work
DMY131 &<br>DMY133	*SCC1-6HA:hphMX4 bar1Δ::natMX4*	This work
DMY3 &<br>DMY4	*hhf1Δ::hphMX4 hhf2Δ::kanMX4 (p413TAR-tet:HHF2) SCC1-6HA:hphMX4 bar1Δ::natMX4*	This work

All strains are isogenic to W303-1A (MATa leu2-3,112 trp1-1 ura3-1 ade2-1 can1-100 his3-11 RAD5)

REFERENCES

1. Ogi H, Wang C-Z, Nakai W, Kawasaki Y, Masumoto H. The role of the Saccharomyces cerevisiae Cdc7–Dbf4 complex in the replication checkpoint. Gene. 2008;414:32–40.

2. Murillo-Pineda M, Cabello-Lobato MJ, Clemente-Ruiz M, Monje-Casas F, Prado F. Defective histone supply causes condensin-dependent chromatin alterations, SAC activation and chromosome decatenation impairment. Nucleic Acids Res. 2014;42:12469–82. *Corrigendum*: Nucleic Acids Research. 2016. doi:10.1093/nar/gkw058.

Table S7b. Oligos used in this study

Name	Sequence	Figure
ECORI +TETH4 F	TTC CGT GTG GCA ATA ATC TC	S2 (left)
ECORI+TETH4 R	TAA CAG GCT TTC ATA CTG CC	S2 (left)
ECORI 508860 F	AGCAGGGATGACTCTGTGAC	S2 (center)
ECORI 509071 R	ATGGTTCAAGGCCGATTGTATG	S2 (center)
HINDIII+TETH4 F	GCA AAT AAA CAA GAA GTT AGC	S2 (right)
HINDIII+TETH4 R	ACG CGT AAC AAT TAC GAA AC	S2 (right)
Up-ERR2pr	TGTTCGTGAATGTGCCTATTGC	3E
Lo-ERR2pr	CGAGCATGCGAGTATTTCTTTTC	3E
Up-IWR1pr	TTCGCAGCATGTGGATTCA	3E
Lo-IWR1pr	TGTGCCGACACAAACAAGAGA	3E
Up-GAT3pr	AAAGAAGCCAGATGAAGTGCCAGG	3E
Lo-GAT3pr	CACCAAATAAGTACTGTGTTAAAGG	3E
Up-tP(UGG)N1	GGCGTGTGGTCTAGTGGTATG	3E
Lo-tP(UGG)N1	CCTCTCGCATGCTTTGTCT	3E
Up-tK(CUU)E1	GGCTCGAACCCCTAACCTTATG	3E
Lo-tK(CUU)E1	GCAATCAATGAACAACCAATGC	3E
Up-CEN3	GTTGAGCA TCCCA TCCAGTT	3E
Lo-CEN3	GGGTAATGGCAAATCTGCTT	3E
Up-CEN5	GTTGCA TTTGCCTTTGGACT	3E
Lo-CEN5	CCCAATTTTAAACGCTCCAA	3E
Up-rDNA	TTTCTGCCTTTTTCGGTGAC	3E
Lo-rDNA	TGGCATGGATTTCCCTTTAG	3E
Up-TEL03L	ATCCCACTACCACATGCCATACT	3E
Lo-TEL03L	AGCATCCGTGTGCGTATGAC	3E
