# Supplementary figures and images for: Crosstalk between chromatin structure, cohesin activity and transcription
Source: Epigenetics Chromatin. 2019 Jul 22;12:47. doi: 10.1186/s13072-019-0293-6 (PMC6647288; doi:10.1186/s13072-019-0293-6)

Figure S1

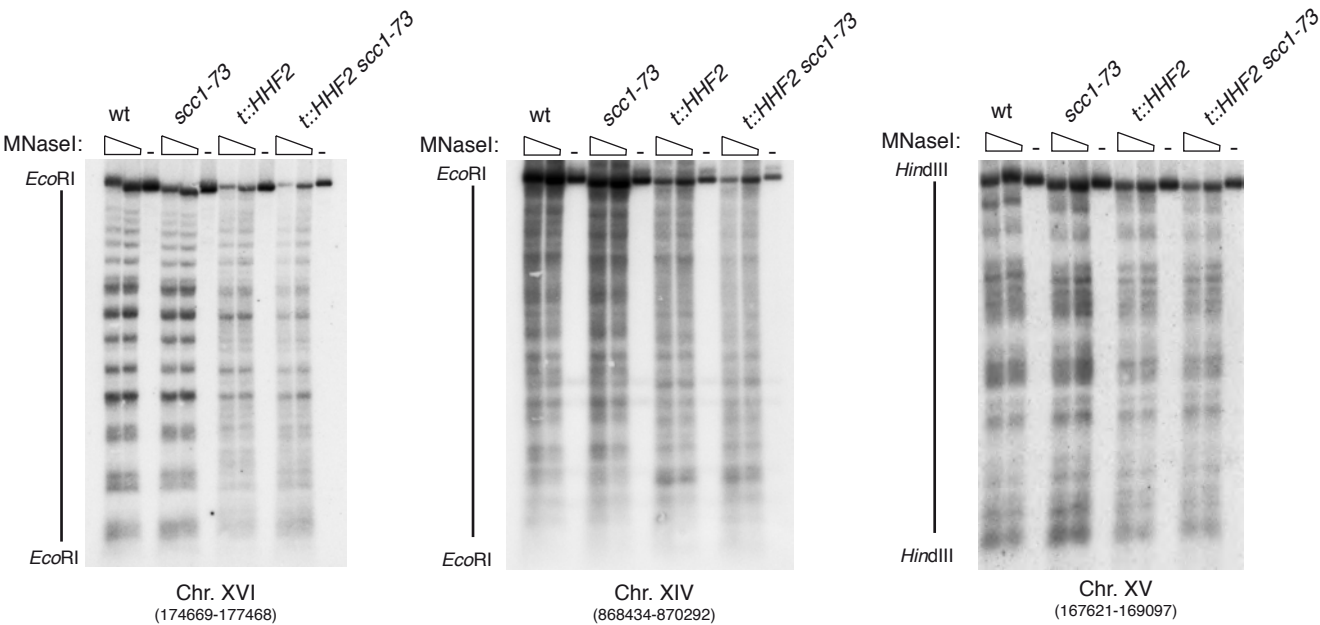

Supplement: Supplementary file 8 — Additional file 8: Fig. S1. Effect of cohesin inactivation in scc1-73 cells on the chromatin structure of three different loci of histone-depleted cells. Nucleosome positioning analyses are shown for the indicated loci after MNase I digestion and indirect-end labeling of the indicated strains synchronized in G1 and released into fresh medium for 1 h at 37 °C until G2/M. [file 13072_2019_293_MOESM8_ESM.pdf]

Figure S3

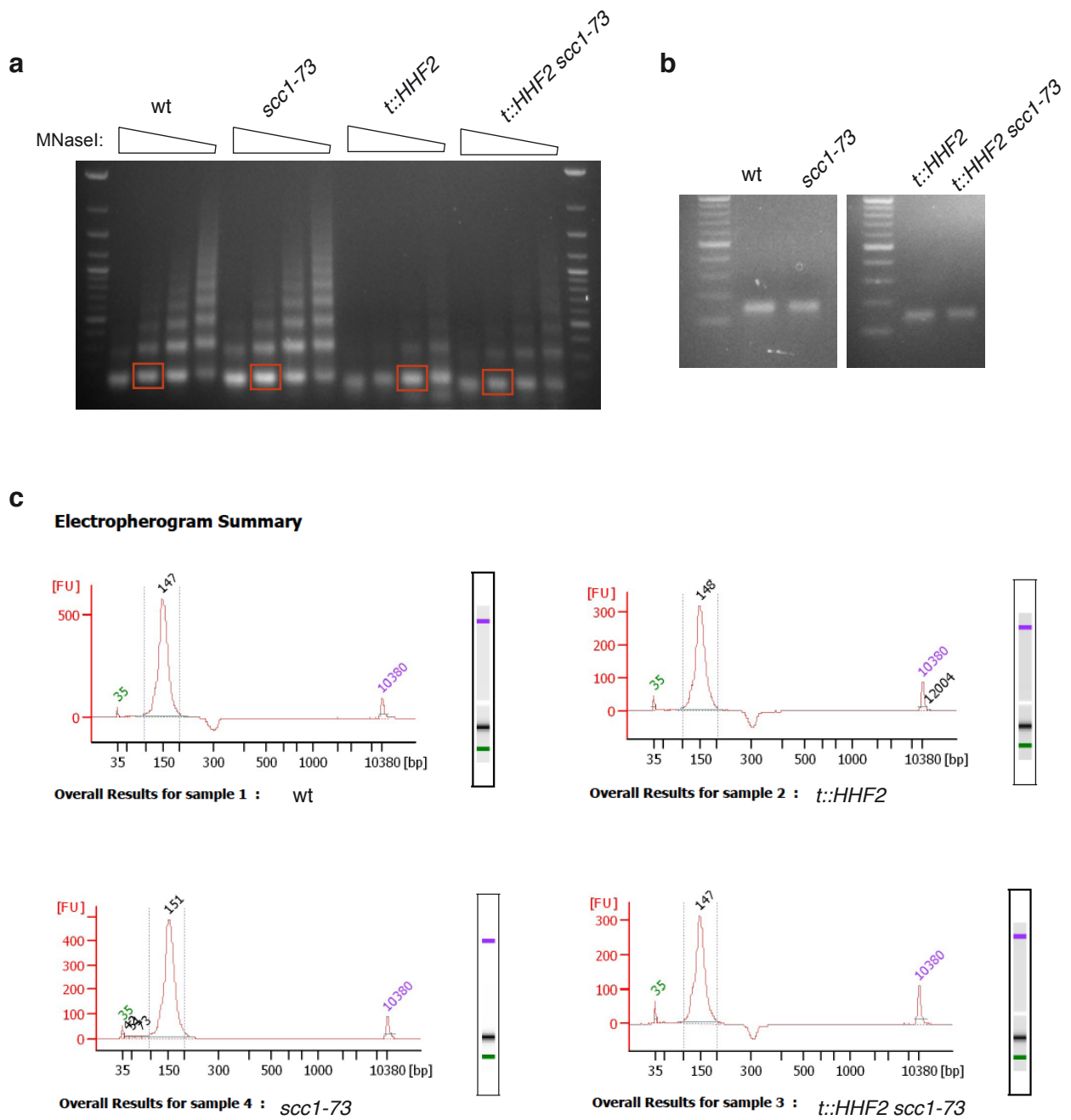

Supplement: Supplementary file 10 — Additional file 10: Fig. S3. Preparation and analysis of the nucleosomal DNA used for MNaseI-seq. a Generation of mononucleosomes in the indicated strains after partial digestions with MNase I. The DNA purified for DNA-seq is marked in red. b, c Electrophoretic (b) and electropherogram (c) analyses of the purified nucleosomal DNA. [file 13072_2019_293_MOESM10_ESM.pdf]
