# Supplementary material for: Crosstalk between chromatin structure, cohesin activity and transcription
Source: Epigenetics Chromatin. 2019 Jul 22;12:47. doi: 10.1186/s13072-019-0293-6 (PMC6647288; doi:10.1186/s13072-019-0293-6)
Supplement: Supplementary file 9 — Additional file 9: Fig. S2. Effect of histone depletion on cohesin binding and distribution. a Cohesin distribution at different regions of chromosomes II and V in wild-type and t::HHF2 cells that have been synchronized in G1 and released into fresh medium until G2/M, as determined by ChIP-on-chip analysis against HA-Scc1. b Probability that IGR that had either lost or gained cohesins after histone depletion would overlap with IGR with altered nucleosomes in t::HHF2 cells or in both t::HHF2 and scc1-73 cells if they were randomly distributed, as determined by a hypergeometric test. The rate between the observed and expected frequencies of common IGR is also shown. The number of IGR in each case is indicated in parenthesis. nd, not determined. [file 13072_2019_293_MOESM9_ESM.pdf]

Figure S2

a

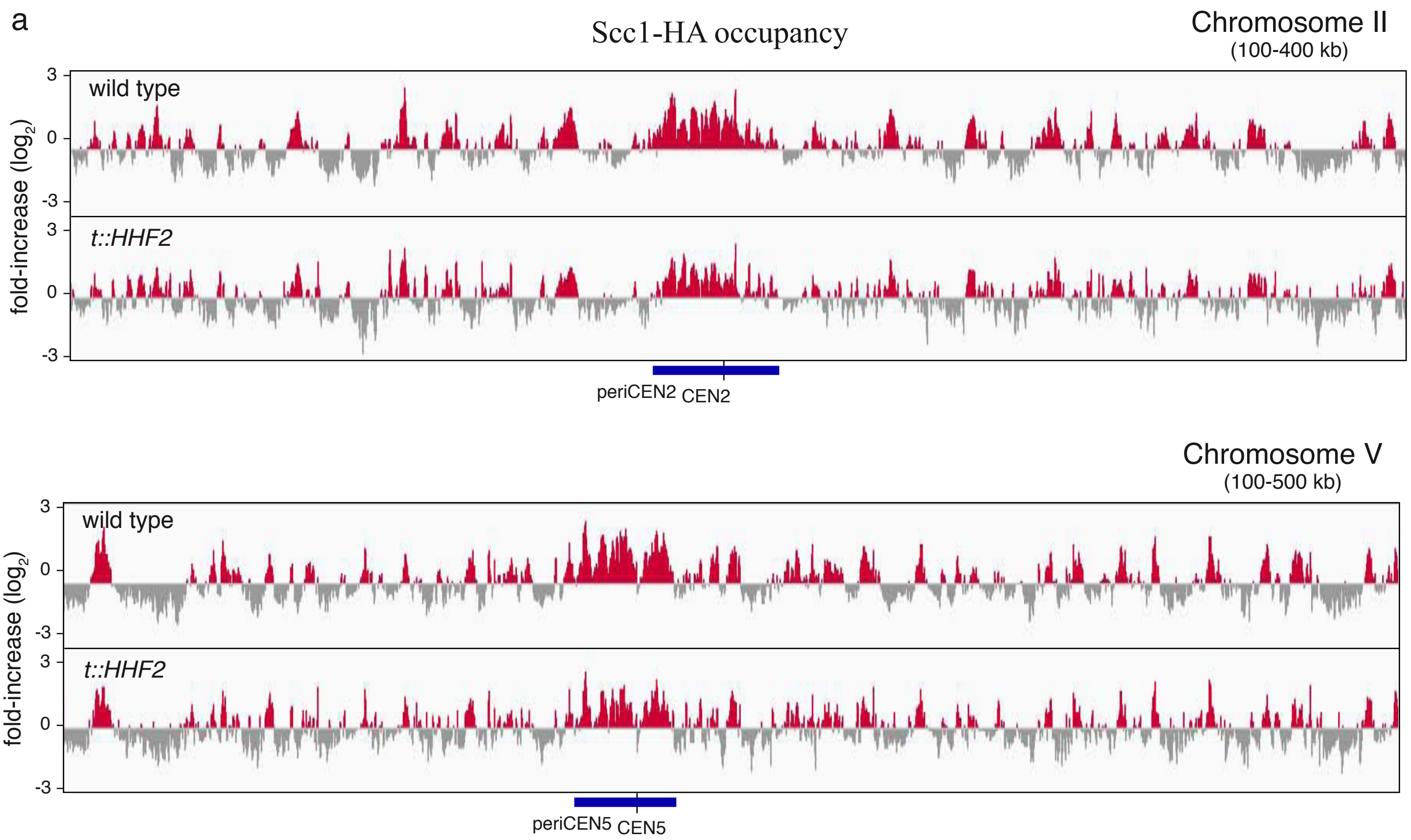

b

IGR with:		up-nuc only in *t::HHF2* (511)	down-nuc only in *t::HHF2* (700)	altered nuc in *t::HHF2* and *scc1-73* (108)
Scc1 lost in *t::HHF2* (465)	*overlap.*	44	43	8
	obs/exp (p-value)	1.22 (0.024)	0.87 (0.04)	1.05 (0.14)
Scc1 acquired in *t::HHF2* (315)	*overlap.*	31	23	nd
	obs/exp (p-value)	1.26 (0.03)	0.69 (0.01)	nd
